# The effect of experienced discrimination and internalized homophobia level in gay and bisexual men on social anxiety level, self-esteem and quality of life

**DOI:** 10.1192/j.eurpsy.2025.578

**Published:** 2025-08-26

**Authors:** B. Yağcı, F. İzci

**Affiliations:** 1 State Hospital, Ministry of Health, İzmir; 2 Training and Research Hospital, Ministry of Health, İstanbul, Türkiye

## Abstract

**Introduction:**

Minority groups in terms of sexual orientation are exposed to specific stressors, unlike the stressors of the general population. Discrimination, stigma, prejudice and violence are more common in minority groups in terms of sexual orientation than heterosexuals, and they affect mental health negatively. Minority stress factors such as perceived discrimination, self-stigmatization and internalized homophobia have negative effects on mental health.

**Objectives:**

External and internal minority stressors, which are associated with social anxiety like many mental illnesses, are also associated with self-esteem and quality of life. Minority stress factors should also be well understood in order to understand the consequences they cause. In this study, it was aimed to examine the relationship between discrimination and internalized homophobia experienced in gay and bisexual men with social anxiety, self-esteem and quality of life.

**Methods:**

85 participants who defined themselves as gay or bisexual man were included in the study.The study is cross-sectional and descriptive, and the participants were reached by the snowball method.Sociodemographic and clinical data form, including the experienced discrimination questions prepared by the researcher, Internalized Homophobia Scale, Libowitz Social Anxiety Scale, Social Interaction Anxiety Scale, Rosenberg Self-Esteem Scale, World Health Organization Quality of Life Scale Short Form Turkish Version (Whoqol-bref Tr) has been applied.The relationship between experienced discrimination in the sample and internalized homophobia; social anxiety, self-esteem and quality of life were examined separately.

**Results:**

It was found a significant relationship between experienced discrimination and social anxiety levels, an inverse relationship was found with self-esteem. A same-way relationship was found between internalized homophobia and social anxiety levels, while an inverse relationship was found with self-esteem. Experienced discrimination and internalized homophobia were both found to be inversely related to quality of life.

**Conclusions:**

In our study, it was found and discussed that experienced discrimination in gay and bisexual men was positively related to the level of social anxiety, and negatively related to self-esteem and quality of life;similarly there was a positive relationship between the level of internalized homophobia and the level of social anxiety, and a negative relationship between self-esteem and quality of life.When these results are evaluated, it is understood that discrimination experiences and negative mental consequences should be taken into account when evaluating homosexual and bisexual men who are minorities in terms of sexual orientation in mental health clinics and practices, psychological support process, preventive mental health practices and policies to be developed.

**Disclosure of Interest:**

None Declared

